# T-Cell Receptors Binding Orientation over Peptide/MHC Class I Is Driven by Long-Range Interactions

**DOI:** 10.1371/journal.pone.0051943

**Published:** 2012-12-14

**Authors:** Mathias Ferber, Vincent Zoete, Olivier Michielin

**Affiliations:** 1 Multidisciplinary Oncology Center, Lausanne University Hospital (CHUV), Lausanne, Switzerland; 2 Swiss Institute of Bioinformatics (SIB), Lausanne, Switzerland; Macquarie University, Australia

## Abstract

Crystallographic data about T-Cell Receptor – peptide – major histocompatibility complex class I (TCRpMHC) interaction have revealed extremely diverse TCR binding modes triggering antigen recognition. Understanding the molecular basis that governs TCR orientation over pMHC is still a considerable challenge. We present a simplified rigid approach applied on all non-redundant TCRpMHC crystal structures available. The CHARMM force field in combination with the FACTS implicit solvation model is used to study the role of long-distance interactions between the TCR and pMHC. We demonstrate that the sum of the coulomb interactions and the electrostatic solvation energies is sufficient to identify two orientations corresponding to energetic minima at 0° and 180° from the native orientation. Interestingly, these results are shown to be robust upon small structural variations of the TCR such as changes induced by Molecular Dynamics simulations, suggesting that shape complementarity is not required to obtain a reliable signal. Accurate energy minima are also identified by confronting unbound TCR crystal structures to pMHC. Furthermore, we decompose the electrostatic energy into residue contributions to estimate their role in the overall orientation. Results show that most of the driving force leading to the formation of the complex is defined by CDR1,2/MHC interactions. This long-distance contribution appears to be independent from the binding process itself, since it is reliably identified without considering neither short-range energy terms nor CDR induced fit upon binding. Ultimately, we present an attempt to predict the TCR/pMHC binding mode for a TCR structure obtained by homology modeling. The simplicity of the approach and the absence of any fitted parameters make it also easily applicable to other types of macromolecular protein complexes.

## Introduction

**Figure 1:**
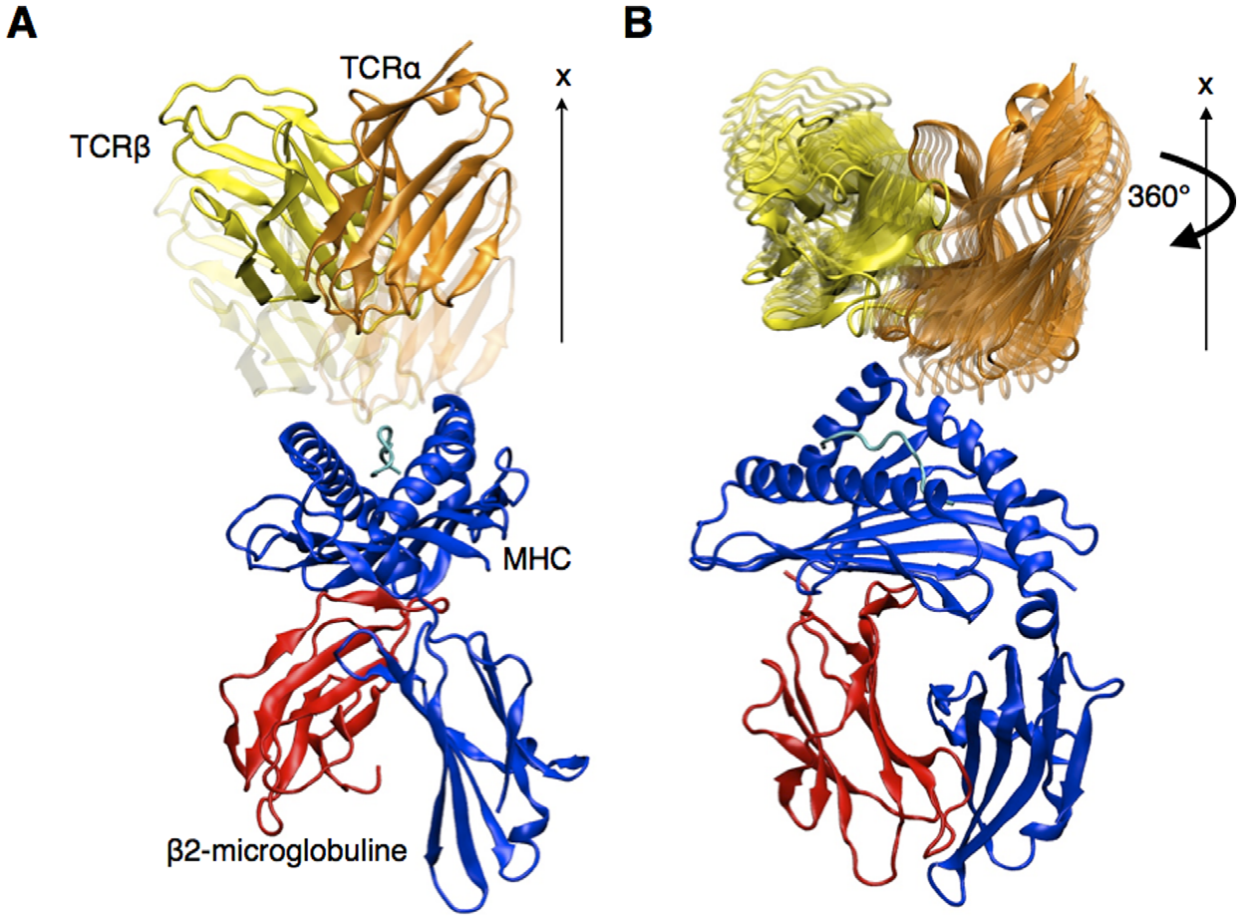
Geometric definition of the TCR binding orientation and rigid displacement protocol. (A) Rigid TCR translation along the x axis. (B) Rigid TCR rotation around the x axis. Rotation step is 5° in this study.

Recognition by the CD8+ T-cell receptor (TCR) of immunogenic peptide (p) presented by class I major histocompatibility complexes (MHC) is one key event in the specific immune response against virus-infected cells or tumor cells, leading to T-cell activation and killing of the target cell [1]. The first determination of the structure of a TCRpMHC complex in 1996 [2] revealed how the molecular recognition of the pMHC by the TCR is mediated by three complementary determining regions (CDR) of each chain the TCR at the interface with the pMHC complex. The CDR1 and CDR2 loops form the outside of the binding site, while CDR3 constitute the central loops in the TCR binding site and mostly interact with the peptide. However, the commonly accepted paradigm of CDR1 and CDR2 binding to the MHC and CDR3 to the peptide does not fully account for the true structural complexity of TCRpMHC complexes and all CDR loops have been shown to interact both with the peptide and MHC [3]–[4]. Over the years, successive releases of TCRpMHC structures have revealed a variety of native TCR binding orientations, defined as the angle that is made between the TCR and the pMHC (Figure 1), depending altogether on the peptide, the MHC and the α/β pairing of the TCR [5]. Recent studies reported TCR/pMHC angles spanning more than 45° variations on the current set of known crystal structures [6].

Understanding the molecular basis that governs TCR orientation over pMHC is still a considerable challenge, and also an important need in the field of TCRpMHC modeling [7] and, as a direct consequence, in the field of rational TCR design and adoptive cell transfer immunotherapy [8]. This questions has been recurrently discussed, but only a few studies have focused on predicting the actual binding mode of given TCRpMHC structures: the study from Varani *et al.* made use of experimental data obtained from NMR chemical shift mapping to obtain lists of buried residues upon binding [9], while the recent study from Roomp and Domingues pedicted the contacts between the pMHC and the TCR, using a training set of TCRpMHC crystal structures [4].

In this work, we use a first-principle based *in silico* approach to uncover the role played by long-range interactions on TCR docking to pMHC. We present a simplified rigid method, which allows scanning quickly the potential orientations of the TCR with respect to pMHC at long distances, and computing the effective energy at each position. This approach was applied to a set of crystal structures to test the agreement between the position of energetic minima and the native binding sites. In 92% of the cases, the 0° minima, corresponding to the native orientation, was the energetically most favorable, demonstrating the predictive-ability of the method. Our scoring scheme, based on the CHARMM force field with the FACTS implicit solvation model [10], allowed the decomposition of the effective energy into residue contributions, and the study of the importance of the different CDR, the MHC and the peptide towards defining the overall TCR orientation. Ultimately, we briefly present and discuss an attempt to predict the TCR/pMHC binding mode using a TCR 3D structure obtained by homology modeling, to asses the efficacy of the approach as a component of a TCRpMHC structural modeling pipeline [7].

**Table 1:** Statistics on TCR rotation profiles.

pdb name	resolution [Å]	TCR	pMHC	primaryminimum [°]	*secondary* *minimum [°]*	polar effective energy difference [kcal/mol]
1ao7	2.6	A6	tax/HLA-A2	0	*175*	4 (lower primary minimum)
1bd2	2.5	B7	tax/HLA-A2	−5	*150*	0.3
1fo0	2.5	BM3.3	PBM1/H2-Kb	−15	*115*	0.8
1g6r	2.8	2C	SIYR/H2-Kb	−10	*145*	2.2
1kj2	2.7	KB5-C20	PKB1/H2-Kb	−25	−*170*	1
1lp9	2	mAH312.2	P1049/HLA-A2	−5	*170*	3.4
1mi5	2.5	LC13	EBV/HLA-B8	10	−*170*	3.2
1nam	2.7	BM3.3	RGYVYQGL/H2-Kb	−15	*180*	2.5
1oga	1.4	JM22	FLU/HLA-A2	−5	−*120*	0.1
2bnr	1.9	1G4	NY-ESO-1/HLA-A2	0	−*160*	1.2
2ckb	3.2	2C	dEV8/H2-Kb	0	*135*	1.4
2e7l	2.5	M6	QLSPFPFDL/H2-LD	25	*160*	1
2esv	2.6	kk50.4	CMV gpUL40/HLA-E1	−10	*165*	3.6
2nx5	2.7	ELS4	EPLP/HLA-B35	−15	−*160*	0
2oi9	2.35	2C	QL9/H2-Kbm3	20	*160*	1
*2ol3* *	*2.9*	*BM3.3*	*PBM8/H2-Kb*	*65*	*115*	*−0.1*
3dxa	3.5	DM1	EBV/HLA-B44	0	*125*	3.5
3e2h	3.8	844.1	QL9/H2-Ld	20	*155*	2.2
3e3q	2.95	M13	QL9/H2-Ld	25	*165*	0.2
3ffc	2.8	CF34	EBV/HLA-B8	5	−*170*	2.1
3gsn	2.8	RA14	HCMVpp65/HLA-A2	−45	−*175*	−0.6
3h9s	2.7	A6	Tel1p/HLA-A2	0	−*115*	6.3
3hg1	3	MEL5	MART-1/HLA-A2	0	−*170*	0.7
3kpr	2.6	LC13	EEYLKAWTF/HLA-B4	5	−*155*	−0.1
3kps	2.7	LC13	EEYLQAFTY/HLA-B4	5	−*160*	0
*3mv8* *	*2.1*	*TK3*	*HPVG/HLA-B35*	−*35*	−*130*	−*1.3*
**pdb name**	**resolution [Å]**	**TCR**	**pMHC**	**primary** **minimum [°]**	***secondary*** ***minimum [°]***	**effective energy difference [kcal/mol]**
unpublished#	–	A6	tax/HLA-A2 (1ao7)	−5	*–*	*–*
1kgc#	1.5	LC13	EBV/HLA-B8 (1mi5)	−10	*–*	*–*
1tcr#	2.5	2C	dEV8/H2-Kb (2ckb)	15	*–*	*–*
2bnu#	1.4	1G4	NY-ESO-1/HLA-A2 (2bnr)	−5	*150*	−0.5
2nw2#	1.4	ELS4	EPLP/HLA-B35 (2nx5)	−15	−*160*	1.2
2vlm#	1.98	JM22	FLU/HLA-A2 (1oga)	10	−*160*	1.4

The 26 TCRpMHC crystal structures of the test set are presented as well as unbound TCR crystal structures tested against their respective pMHC.

* outlier structures.

# unligated TCR.

*–*steric clash.

## Methods

### Default Procedure

In this work, the default procedure can be summarized as follows (see Figure 1). For each crystal structure of TCRpMHC complex, the TCR is translated 8Å away from the pMHC, then rotated using 5° steps. The effective energy of the whole system is computed at each step, and plotted against the TCR/pMHC angle. The plots present the energy variation upon rotations of 360° and are referred to as rotation profiles.

**Figure 2:**
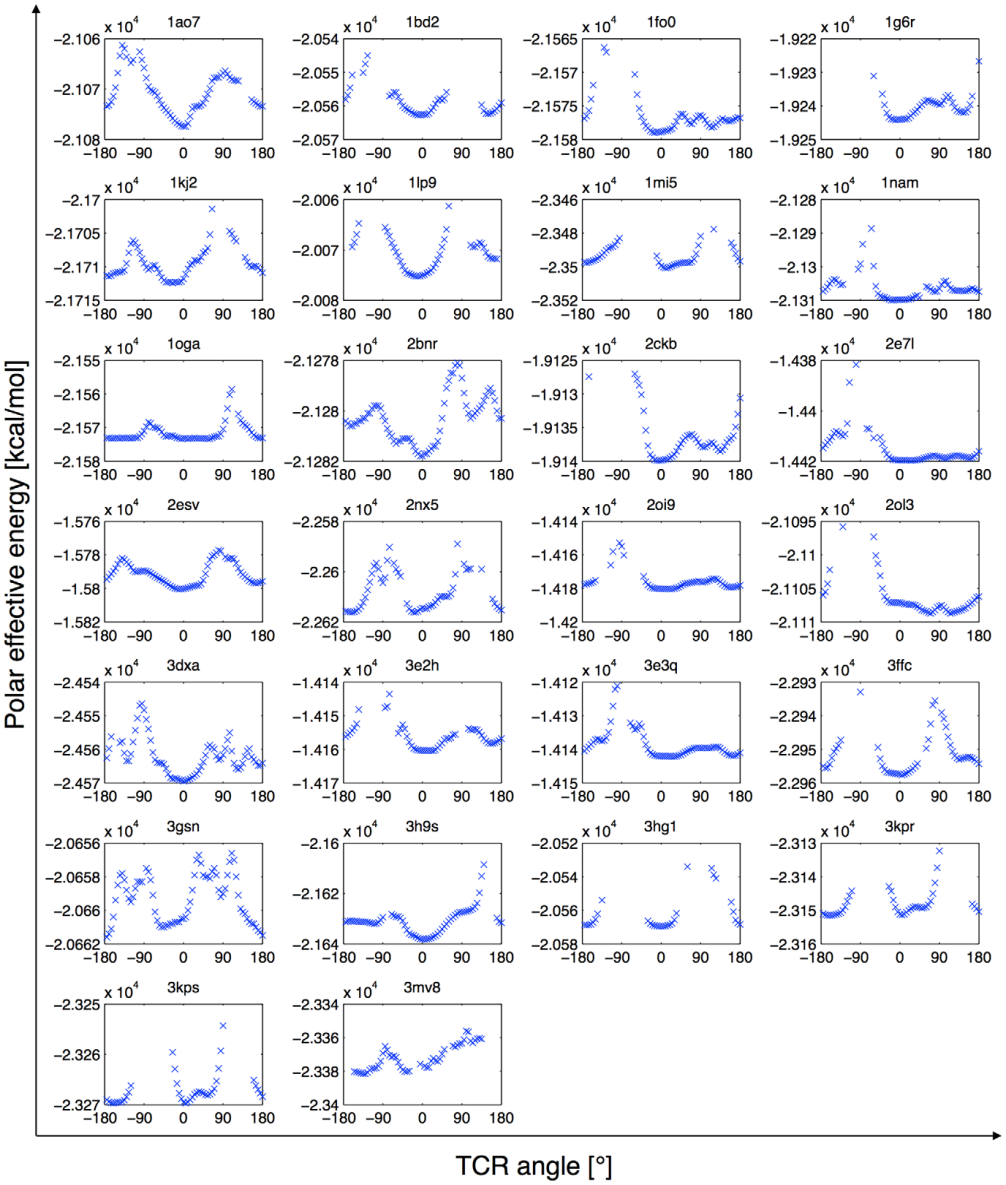
TCR rotation profiles of the test set. The polar contribution to the effective energy of the TCRpMHC complex is plotted against TCR rotation angle around the x axis, after an 8Å translation away from the pMHC. Positions that make steric clashes are ignored.

#### TCRpMHC complexes

26 crystal structures of TCRpMHC class I have been selected in the MPID-T2 database as of July 2011 (http://biolinfo.org/mpid-t2) [11] and were downloaded from the PDB [12]. They are listed in Table 1. Redundant structures, such as those bearing identical chains with point mutations, were not selected. Structures bearing non-natural peptides or peptides longer than 11 residues were excluded as well. All calculations were performed on systems consisting in the peptide bound to MHC, the β2-microglobulin, and the variable domains of the TCR α and β, with the exception of the 2e7l, 2esv, 2oi9, 3e2h and 3e3q systems for which only the binding site of the MHC (residue 1 to 175), the peptide and the TCR were available in the crystal. In the following, the complex formed by the peptide, the MHC class I, and the β2-microglobulin (if available) is simply designated as peptide-MHC (pMHC).

**Figure 3:**
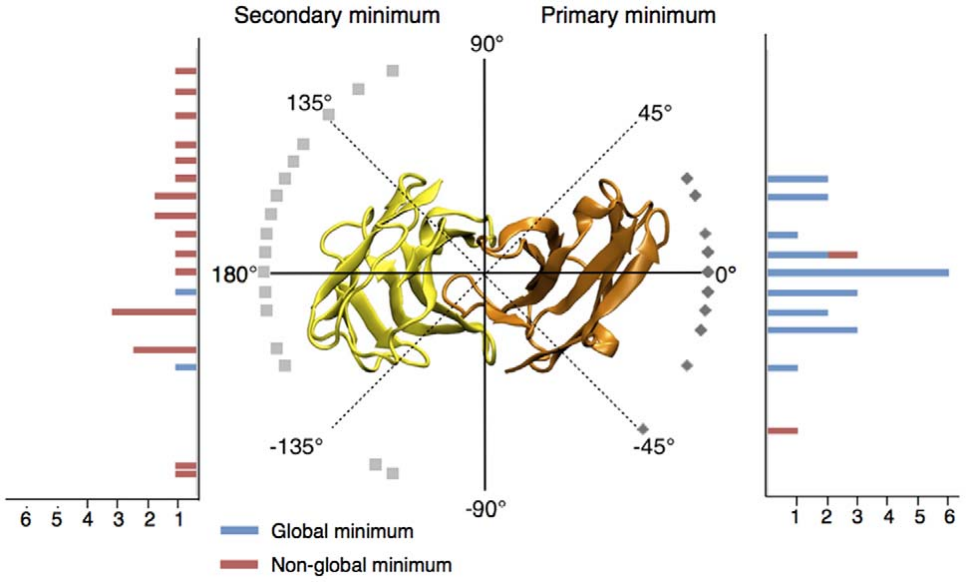
Summary of the repartition of primary and secondary minima in TCR rotation profiles. 0° corresponds to the native orientation of each bound conformation. The positions of the primary minima are shown on the right half of the circle and reported on the right side histogram, which indicates the number of occurrences of each minimum in the test set. Secondary minima are similarly shown on the left half of the figure. The color code of histograms discriminates between global (blue) and non-global (red) minima. Outliers are not displayed.

Additionally, 8 TCRpMHC class II structures were similarly selected in the MPID-T2 database. The results obtained from this set of structures are described in the supplementary materials. In the following, MHC stands for MHC class I, unless specified otherwise.

**Figure 4:**
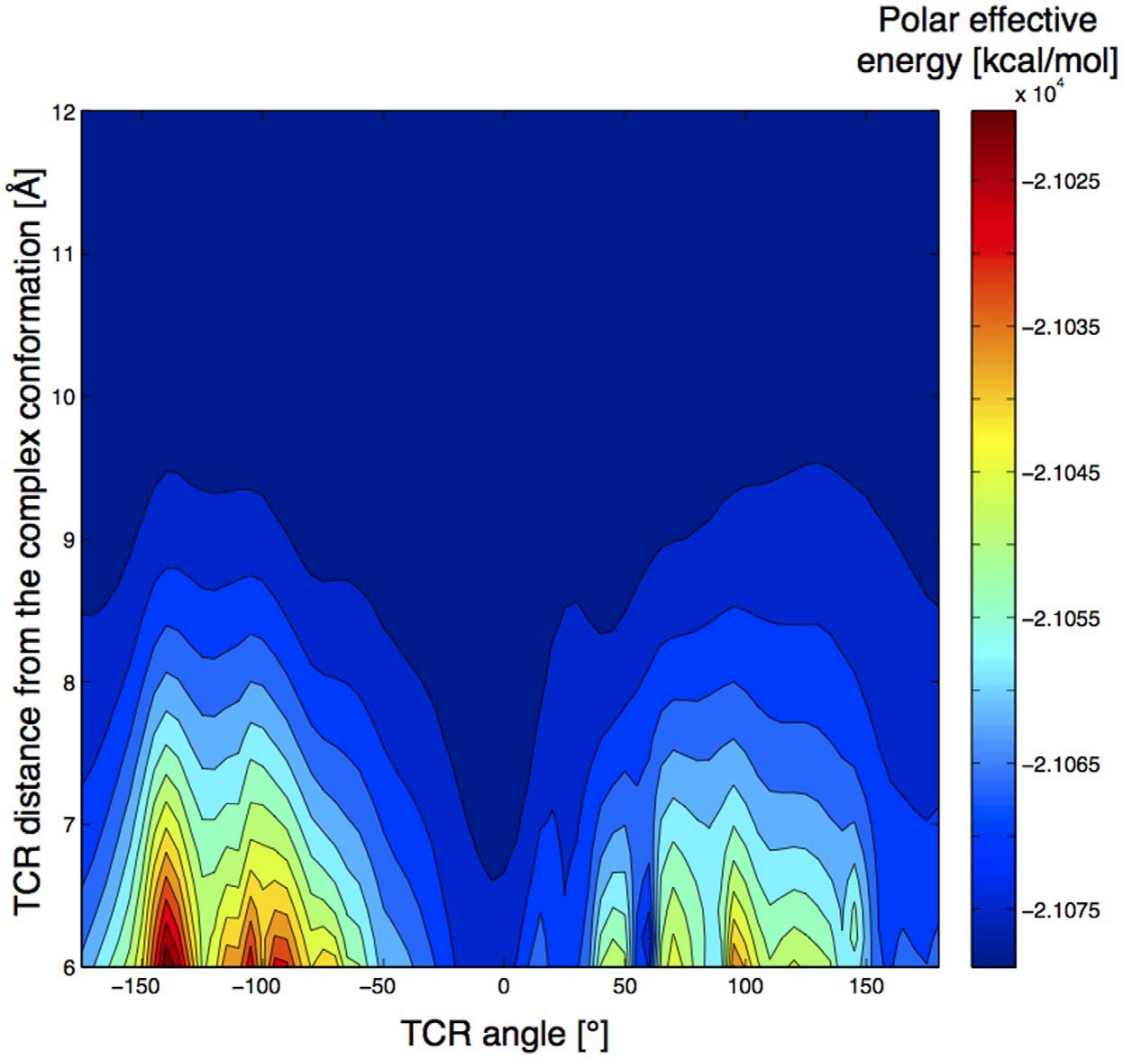
Landscape representation of the evolution of TCR polar energy rotation profiles of 1ao7 as a function of the TCR/pMHC distance. The energetic preference for the native orientation (0°) is clearly visible. Rotation profiles were not computed at distances lower than 6Å due to the numerous steric clashes below that distance.

#### Force field

All calculations were handled by the CHARMM program version c35b1r1, using the CHARMM22 all-atoms force field. First, the His residue protonation states were determined and the systems were set up for use with CHARMM according to an in house automated procedure (V. Zoete, private communication) that is also used behind the SwissDock small molecule docking web service [13]. Following this setup, the system was minimized by 500 steps of steepest descent, using the Fast Analytical Continuum Treatment of Solvation (FACTS implicit solvation model) [10]. Default parameters were used, including a dielectric constant of 1.0 for the protein and 80 for the solvent. A shifting function was applied on the electrostatic with a 12Å cutoff. Finally, the internal degrees of freedom of the TCR and the pMHC were frozen during the remaining calculations (e.g. rigid bonds, angles and dihedral angles), unless specified otherwise.

**Figure 5:**
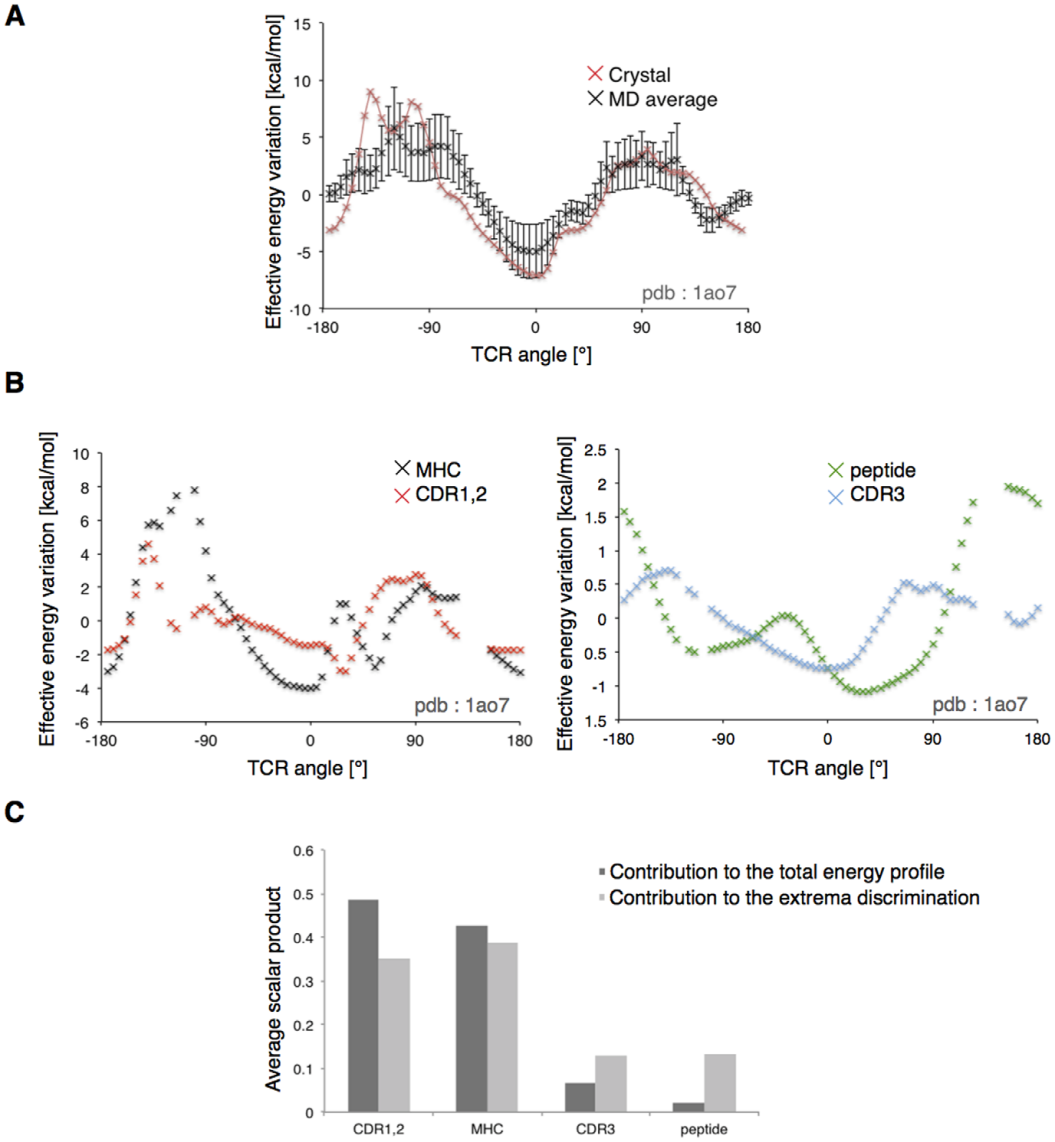
Robustness and decomposition of the rotation profile. (A) Comparison between the 1ao7 rotation profile (red) and the average of the 40 rotation profiles calculated after A6 TCR extracted from MD. Vertical bars are the standard deviations at each position. (B) Decomposition of the rotation profile of 1ao7. The polar effective energy is separated into contributions of the MHC (black), the CDR1,2 (red), the peptide (green) and the CDR3 (blue). (C) Average correlation coefficients of subgroups rotation profile regarding the rotation profile of the whole system. The CDR1,2 and MHC are responsible for 92% of the signal. The CDR3 and peptide are more important for discriminating the native from the opposite orientation.

#### TCR space exploration

The Cartesian axes were oriented along the principal axes of the TCR molecule (Figure 1). The x axis was defined as the principal axis of the TCR, as calculated by CHARMM. This axis is perpendicular to the plane of interaction with pMHC. The TCR space exploration consisted in a rigid displacement (translation or rotation) of the TCR to successive positions, from which the long-range interaction score values were calculated. First, the TCR was separated from the pMHC by translating it along the x axis, allowing rotations around the x axis without steric clashes. We calculated the electrostatic effective energy after each translation and rotation, as described below.

**Table 2:** Summary of correlation coefficients of the sub-systems CDR1,2, MHC, CDR3 and peptide, for each crystal structure.

	1ao7	1bd2	1fo0	1g6r	1kj2	1lp9	1mi5	1nam	1oga	2bnr	2ckb	2e7l	2esv	2nx5
CDR1,2	0.286	0.308	0.635	0.462	0.331	0.3	0.333	0.722	0.618	0.065	0.399	0.709	0.87	0.398
MHC	0.624	0.504	0.376	0.376	0.657	0.481	0.523	0.278	0.382	0.206	0.513	0.221	0.006	0.616
CDR3	0.081	0.198	−0.008	0.009	0.001	0.182	0.057	0.001	−0.002	0.719	0.007	0.027	0.137	−0.023
peptide	0.009	−0.009	−0.004	0.152	0.011	0.037	0.087	−0.001	0.002	0.01	0.081	0.043	−0.013	0.008
	**2oi9**	**2ol3** *	**3dxa**	**3e2h**	**3e3q**	**3ffc**	**3gsn**	**3h9s**	**3hg1**	**3kpr**	**3kps**	***3mv8*** *		**average**
CDR1,2	0.794	0.633	0.562	0.394	0.809	0.183	0.421	0.27	0.282	0.772	0.734	*0.084*		0.48
MHC	0.206	0.335	0.431	0.411	0.17	0.806	0.57	0.662	0.712	0.207	0.273	*0.054*		0.41
CDR3	−0.002	0.047	0.043	0.162	0.005	0.005	0.008	0.012	0.005	−0.004	−0.03	*0.219*		0.07
peptide	0.001	−0.015	−0.035	0.032	0.016	0.005	0	0.056	0	0.026	0.022	*0.643*		0.04

* outlier structure.

#### Effective energy calculation

The effective energy of the TCRpMHC system in a given state is described as the sum of the intramolecular energy and the solvation free energy:
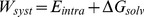
Where the intramolecular energy of the system is the sum of the bonded energy 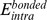, the van der Waals energy 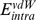, and the electrostatic energy in vacuum 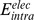. The solvation free energy is the sum of a polar term 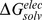 and a non-polar term 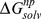. The effective energy can be rewritten as follows:



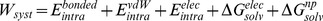



**Figure 6:**
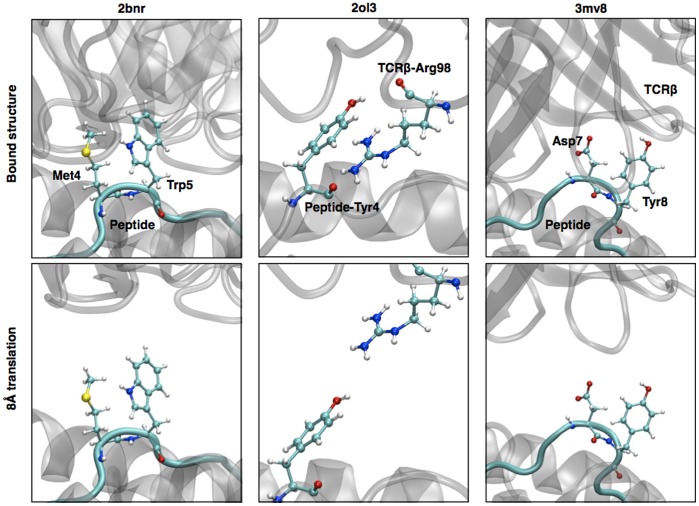
3D structure of the bound and the translated positions of the TCR for the three outlier complexes. The incriminated residues are highlighted in ball and stick representation.

Since the TCR and the pMHC were kept rigid, 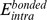 is constant and was neglected in the following. Also, unless specified otherwise, TCR and pMHC were always separated by at least 8Å, so that 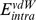 and 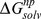 were found constant. As a result, all effective energy variations could be calculated from:
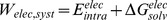



The 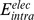 term was calculated as the sum of coulomb interactions, while the electrostatic solvation energy 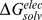 was calculated using the FACTS implicit solvation model [10]. Default parameters were used, as explicited earlier.

**Figure 7:**
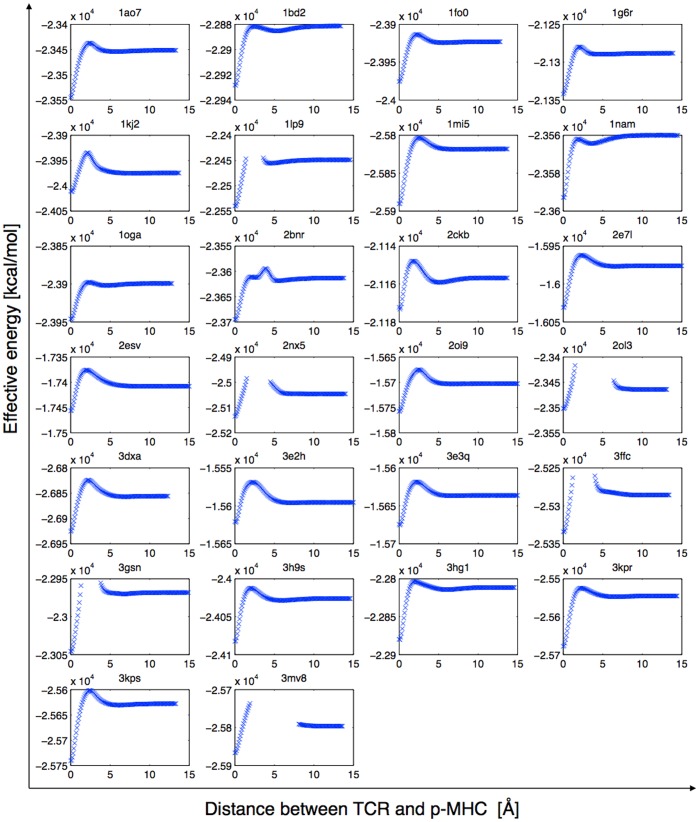
Rigid pulling profiles of the test set. The whole effective energy (see methods) is plotted against TCR translation away from the pMHC. Positions that make steric clashes are ignored.

#### FACTS energy decomposition

The coulomb and the electrostatic solvation energies were decomposed as described previously [14]. The contribution of the atom i to the total coulomb energy of the system is given by
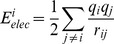
where j loops over all the atoms of the system, and r_ij_ is the distance between atom i and atom j bearing the charges q_i_ and q_j_, respectively. Since the FACTS approach makes use of the Generalized Born formula [15] to calculate the electrostatic part of the solvation energy, we calculated the contribution of the atom i using [14]:



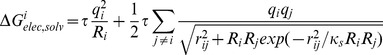
where 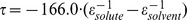, considering energies expressed in kcal/mol and distances expressed in Angstroms. R_i_ and R_j_ are the FACTS Born radii. The Born radius of atom i was calculated using CHARMM as follows. First, the charge of every atom of the system is set to zero, except for atom i of charge q_i_. The FACTS electrostatic solvation energy of the system - thus corresponding to the electrostatic solvation energy of atom i in the context of the uncharged protein, 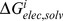 - was then calculated. The Born radius was finally obtained according to its definition [10]:



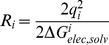



**Figure 8:**
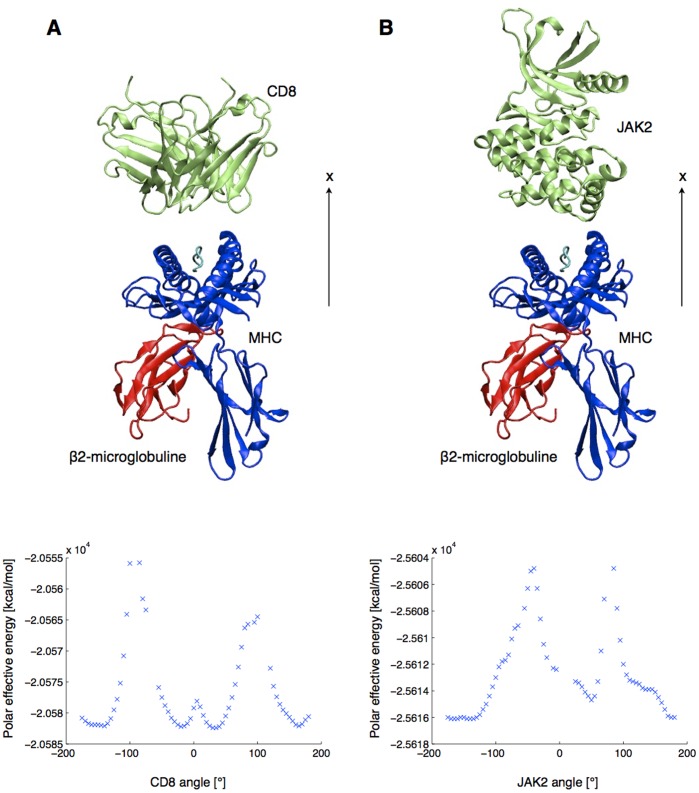
Starting position and rotation profiles of two unrelated proteins (lime green) in front of the pMHC. (A) CD8 homodimer. (B) JAK2 tyrosine-protein kinase.

The approach is similar to MM-GBSA decompositions of former studies [16] although the current FACTS implementation in CHARMM c35b1r1 requires the computation of the Born radii of each atom in the system. The FACTS model was preferred to other implicit solvation models in this study, since it is as efficient in reproducing Poisson Boltzmann solvation energies as GB-MV2, while being 10 times faster. Also, contrarily to GB-MV2, FACTS is not a grid-based method and therefore does not show any unphysical energy variations upon rigid rotation of a protein (see Figure S2).

**Figure 9:**
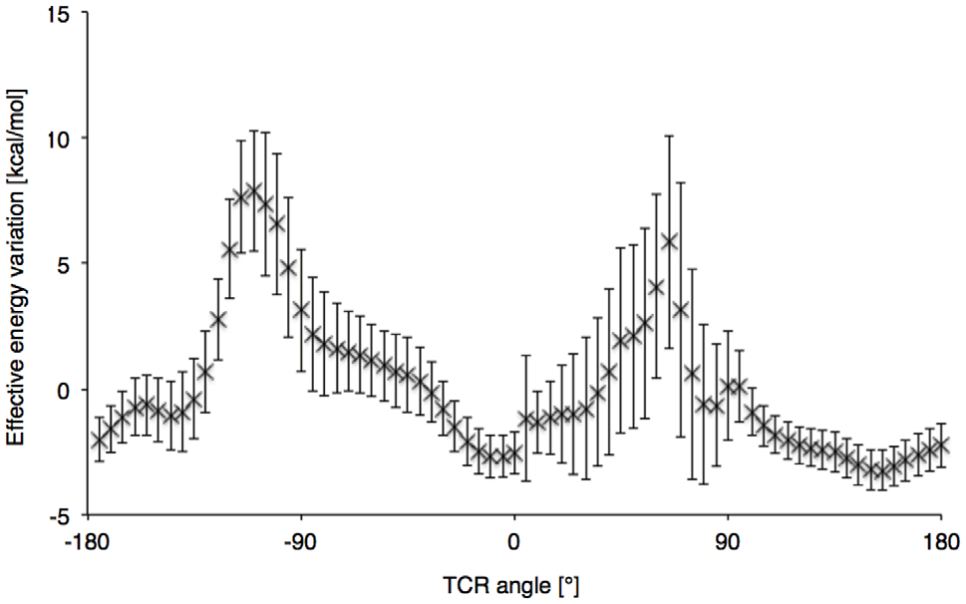
Average rotation profile of 500 A6 TCR modeled by homology. Vertical bars are the standard deviations at each position.

#### Correlation coefficient

The rotation energy profile vector, 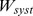, is defined as a collection of n values of the effective energy of the system, calculated for n angle values regularly distributed along a 360° revolution of the TCR around the x axis:
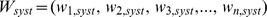



In this work, considering 5° rotation steps, n is always equal to 72. For each of the n TCR positions, the energy of an atom selection was stored into a vector *W_sel_* :
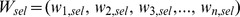



Considering the following average:
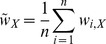
where X stands for “syst” or “sel”, we defined the normalized and centered vector as:



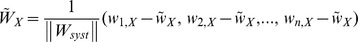



Therefore, the contribution of an atom selection to the profile of the effective energy variation as a function of the TCR orientation was estimated by calculating the correlation coefficient:
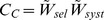



The correlation coefficient is dimension-less, and takes values between −1 for totally anti-correlated vectors and 1 for totally correlated vectors.

#### Molecular dynamics setup

Molecular Dynamics simulations were performed in Gromacs 4.5.1 with the CHARMM27 force field [17], on the A6 TCR extracted from the PDB structure 1ao7. The TCR was solvated in an orthorhombic periodic box of TIP3P water molecules [18], resulting in a system size of 74.2×63.4×48.5 Å^3^ for a total of around 70000 atoms. During the dynamics, the Lennard-Jones interactions were treated with a switch function reaching zero at 14Å, and the electrostatic interactions were calculated using the particle-mesh Ewald (PME) method. After energy minimization, the system was heated up to 300 K during 100 ps with positional restraints on all heavy atoms. The system was subsequently equilibrated at constant temperature and volume during 200 ps, then using a Berendsen temperature and pressure coupling for 200 ps [19], and ultimately using two separate Nosé-Hoover thermostats [20]–[21] for the solvent and the protein, as well as a Parrinello-Rahman barostat [22], for 400 ps. This last setup was conserved during the main trajectory, during 4 ns, ensuring the correct conservation of the NPT ensemble.

#### TCR homology modeling

500 models of the A6 TCR [23] were built using the homology module of the TCRep 3D approach, as described elsewhere [7]. TCRep 3D makes use of state of the art homology modeling using the Modeller 9v5 software [24], complemented by the use of additional dihedral restraints applied on CDR 1 and CDR2 to drive the loops towards their canonical conformations [25]. The templates were selected in our set of TCR (see above), excluding those bearing similar α or β chains.

## Results

We assessed the variations of the effective energy of the system upon rigid motions of TCR relative to pMHC. The calculations were made on a set of 26 TCRpMHC complexes, corresponding to all non-redundant systems whose experimental structure has been determined (see methods). The TCR was moved 8Å away from the pMHC molecule, along its principal axis, orthogonal to the TCR/pMHC interaction plane (Figure 1A). Subsequently, 5° rotation steps around this axis were successively applied to the TCR until a complete revolution was obtained (Figure 1B). At each position, the sum of Coulomb interactions and the electrostatic solvation energy, obtained with the FACTS implicit solvation model [10], was computed.

### TCR Orientation and Effective Energy Minima

Figure 2 shows the variations of the effective energies during the rotations of the TCR with respect to pMHC. At each position, the smallest distance between the two parts was identified and the van der Waals interaction between the two corresponding residues was computed to verify that they did not clash. Positions where TCR clashes with pMHC were ignored. 0° corresponds by definition to the native orientation seen in the X-ray structure. As can be seen, the rotation profiles are mostly characterized by local minima near the 0° and the 180° positions and sharp maxima near the 90° positions. These rigid profiles suggest that, at an early stage of the TCR approach, long distance interactions with pMHC already play a significant role to guide TCR orientation. The local minima near the native and the opposite orientation were defined as the primary and secondary minimum, respectively, and were recorded in Table 1.

Considering the energy profiles, the structures 2ol3 and 3mv8 were considered as outliers and were analyzed separately (see below). Interestingly, the primary minimum is the global energy minimum in a large majority of profiles (for 22 over 24 complexes). On average, it is distant by only 11.0° (SD = 11.2) from the native orientation, and ranges from −45° to 25° (Figure 3). Noticeably, 20 profiles showed an energetic minimum located at less than 20° from the native orientation. Following the primary translation of 8Å, the amplitude of the energy variation along the TCR revolution ranges from 3.0 kcal/mol for 2bnr to 37.9 kcal/mol for 2e7l, with an average of 19.3 kcal/mol (SD = 8.3) over the 24 profiles after neglecting the TCR positions that make steric clashes. We also investigated the dependency of the rotation energy profiles on the distance between TCR and pMHC. Figure 4 illustrates how the amplitude of the signal increases when the TCR/pMHC distance decreases from 12Å to 6Å, and how the TCR/pMHC approach can be guided by an energetic funnel that will lead the two partners in their final native complex orientation.

The same study was performed on 8 TCRpMHC class II structures. The resulting rotation profiles showed similar shapes and well defined primary minima at 6.3° (SD = 2.3) on average (see Figure S1).

### Reliability of the Rigid Approximation

In the rigid body approximation, we neglected willingly the dynamic properties of both the TCR and the pMHC, such as internal fluctuations, and possible structural rearrangement of the system during the binding [26]–[27]. We used Molecular Dynamics (MD) simulations of the TCR to verify that the nature of the long-distance interactions between TCR and pMHC observed with a rigid model are not significantly changed by the structural fluctuations accessible at room temperature. Thus, we extracted 40 distinct conformations of the unbound A6 TCR (PDB ID: 1ao7), one every 100 ps along a 4 ns MD simulation trajectory. Rigid TCR rotations were performed using these conformers placed at 8Å from the crystal conformation of the Tax/HLA*A0201 pMHC (PDB ID: 1ao7). Importantly, the average energy profile shows a shape similar to the one calculated using the X-ray conformer (Figure 5A). The averaged primary minimum was indeed situated at −5.39° (SD = 8.73), from the native orientation, while it was predicted at 0° using the crystal structure. Clearly the shape of the TCR rotation profiles does not depend on the detailed atomic coordinates of crystal structures. This suggests that the role of long distance interactions observed in the rigid body approximation also holds in the dynamical process of TCR approach towards pMHC.

Additionally, rotation profiles were computed for the available unbound TCR structures. The 6 TCR where placed at 8Å from the crystal conformation of the corresponding pMHC in the bound TCRpMHC structures (Table 1). Again, we identified well-defined primary minima at less than 15° from the native orientation.

### Energy Decomposition

The contributions of structural sub-groups to the TCR rotation energy profiles were calculated by performing FACTS energy decomposition. The approach is similar to MM-GBSA binding free energy decompositions of former studies [16]. We considered 4 distinct parts of the system: MHC, peptide, CDR3, and the TCR without CDR3. The aim was to assess the importance of a selected region of the system in the definition of the energetic minimum, which defines in turn the path that leads the TCR towards its bound conformation. As illustrated on Figure 5, with the 1ao7 complex, a typical decomposition resulted in large contributions from the MHC helices and from the CDR1 and 2 of the TCR (see also Figure S3). Correlation coefficients between the sub-group and the entire system rotation energy profiles (see Methods) confirmed this on 24 structures (Table 2).

The noticeable outliers are discussed below.

#### 2bnr

This structure is formed by the 1G4 TCR bound to the NY-ESO-1 antigen. The peptide is characterized by a central Met-Trp pair pointing out, towards the TCR [28]–[29] (Figure 6). Even at 8Å from the bound conformation, the side chains of the peptide remain close enough from the CDR3 for this interaction to play a preponderant role upon rotation of the TCR. The structure remained in good agreement with the shape of most of the rotation energy profiles (Figure 2), with well-defined primary and secondary minima.

#### 2ol3

The rotation profile of this structure failed to discriminate between two opposite minima. Two local minima were found close to 90° from the native orientation (Figure 2). In the bound conformation, the residue Arg 98 of the CDR3β is deeply buried under the Tyr4 of the peptide [30]. In our rigid approximation, after the 8Å translation, the two side chains face each other at a distance lower than 3Å in an unfavorable conformation (Figure 6). By removing the contributions of these two residues, we obtained a rotation profile with a well defined primary minima located 10° away from the native orientation (see also Figure S3).

#### 3mv8

The peptide in this structure is a particularly long EBV peptide (11 residues) in a bulged conformation, bearing two side chains (Asp7 and Tyr8) that are deeply buried inside the TCRβ chain [31] (Figure 6). These two residues play a disproportionate and unrealistic role in the TCR rotation profiles at 8Å. Clearly the rigid body approximation is unsuited for this structure. Interestingly, by ignoring the contribution of these two residues, we dramatically improved the rotation profile quality (Figure S3).

On average, 92% of the rotation energy profile is carried by the periphery of the binding site (Figure 5C), suggesting that the interaction of MHC with CDR1,2 is mostly responsible for guiding the TCR towards the native orientation. This observation is in fair agreement with current knowledge regarding TCR germline bias for MHC [32]. Finally, by considering only the rotation profiles in a 60° range around the primary and secondary minima, the computation of correlation coefficients resulted in an increased role of the CDR3-peptide, from 8% to 26% of the contribution (Figure 5C), showing that the interaction between CDR3 and the antigen helps discriminating between the native and the opposite orientation during TCR approach. At 8Å, except for the 2ol3 structure (see above), the native orientation was indeed always preferred by the CDR3-peptide interaction by an energy difference of 2.38 kcal/mol (SD = 2.90), on average.

### Rigid Pulling

Additionally, we performed a rigid body undocking of the TCR, starting from the complex structure. The full effective energy of the system, including the van der Waals energy 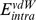 and the non polar term of the solvation energy 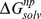 (see methods), was calculated every 0.1Å along the principal axis. As shown on Figure 7, the energy profiles clearly show the typical energy barrier of the TCR binding to pMHC [26]. The estimated binding free energy values in that approximation are comprised between −133 kcal/mol and −15.5 kcal/mol, which is in reasonable agreement with TCRpMHC binding free energies estimated *in silico* using the MM-GBSA method [14], considering our rigid approximation, and neglecting the entropy terms and 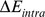 the variation of the internal energy upon reorganization. Interestingly, for all complexes, the energy barrier was identified near 5Å from the bound position. Our results showed that long-range interactions do have an impact on TCR orientation towards MHC at longer distances. This suggests that the orientation driving force is indeed distinct from the final approach and induced fit mechanisms.

### Negative Controls

We investigated the shape of rotation profiles of non TCR proteins. We selected the crystal structures of a CD8 homodimer (PDB ID : 1akj) and a JAK2 tyrosine-protein kinase (PDB ID : 3ugc). The former was selected to compare the TCR rotation profiles with another type of Ig-folded dimer and the latter to test a monomeric structure whose fold is unrelated to that of the TCR. The two systems were protonated with CHARMM, minimized (see methods), and aligned in front of the pMHC (PDB ID: 1ao7) in order to share the principal axes of the TCR (Figure 8). First, the rotation profile of the CD8 showed the same two effective energy maxima close to 90° and −90°. However, the 0° and 180° positions are local maxima in this case, surrounded by local minima situated at −160°, −20°, 30° and 165°. The very symmetrical shape of the profile can be explained by the homodimeric nature of the CD8. Second, the rotation profile of JAK2 showed no symmetric tendency, with two well-defined maxima distant from only 125°. This illustrates that the rotation profiles reported in Figure 2 are specific to TCR with respect to the pMHC.

### Using TCR Structures Obtained by Homology Modeling

500 structures of the A6 TCR were obtained using homology modeling. We calculated an average heavy atoms RMSD of 2.01Å (SD = 0.05) between the models and the A6 crystal structure, after optimal least square structural alignment. Each model was minimized by 500 steps of steepest descent. The TCR of the crystal structure (PDB ID: 1ao7) was successively replaced by the different homology models at 8Å from the crystal conformation, and rigid rotations were performed in front of the MHC. The obtained average rotation profile is shown in Figure 9. As can be seen, the two minima were again visible, and the primary minimum was situated at 12.2° (SD = 16.0) on average. This illustrates the potential of our rigid approach for docking predictions in combination with a TCRpMHC structural modeling pipeline [7].

## Discussion

The aim of this study was to explore, using a first-principle based approach, the long-distance driving force that guides TCR in the proper orientation with respect to the pMHC. The assumption regarding the existence of such driving force was based on general knowledge regarding the binding process of two distinct proteins [33]–[34]. The binding mode of the TCRpMHC is indeed essential to predict and understand the peptide recognition leading to T-cell activation. Furthermore, a large diversity of orientations has been seen in the experimental complexes, for the various TCR and pMHC.

### TCR Binding Mechanism

The dynamical mechanism the association of the TCR with the peptide-MHC until the final binding mode has been discussed intensively in the literature [26] [35]. In the meantime, knowledge about the geometry of TCRpMHC interaction was recently extended [5] [32]. Current models for TCR/pMHC association describe a two steps approach, where the CDR1 and CDR2 loops first scan the MHC molecule to form, in turn, specific contacts and define the general orientation of the TCR over pMHC (association step). A second step (stability step) includes specific interactions and induced fit of the peptide with CDR3 [26]. The prevalent role of CDR1 and 2 in the definition of the binding mode was also mentioned by Garcia *et al.* [32] who stated that CDR1,2/MHC interaction defines the general footprint of the TCR on pMHC, while the CDR3 and the peptide are only responsible for subtle orientation variations. Finally, the study by Collins and Riddle [5] proposes a model where the binding of the CD8 to the MHC, while necessary for TCR signaling, is subsequent to the definition of the docking orientation itself. In this model, the binding of CD8 could be regarded as a mechanism that helps discriminating between the native and the opposite orientation of the TCR. However, it is not supposed to be determinant to define the precise binding orientation.

In our approach, TCR rotation energy profiles revealed that the native binding mode orientation is defined at an early stage of the TCR approach, before the emergence of a direct contact between CDR1/CDR2 and pMHC. Before the appearance of the binding energy barrier, the native orientation was already predicted with a deviation of 11° in average, using only a model of long-range interactions, which is quite satisfying in view of the 45° amplitude in the native binding mode orientations observed on crystal structures [6].

The energy decomposition was performed using a generalized Born model, using the method described in a previous study of TCR-p-MHC binding [14]. In the latter, it was shown that such a decomposition approach gives results that are closely related to those of a computational alanine scanning, when used to assess the contributions of single residues to the binding free energy. The decomposition confirmed the importance of the CDR1,2/MHC interaction for the TCR/pMHC orientation, since it defines 91% of the signal of rotation energy profiles, on average (see Results). This contribution successfully delineates a primary minimum energy orientation that leads to the final binding mode and is also capital for preventing orthogonal binding. This result supports strongly the observation from Khan and Ranganathan [6], who identified a ring of charged residues at the pMHC interface, which interacts with CDR1 and CDR2 with complementary charges. We reported that the role of CDR3 and peptide residues is of lesser importance at long distance (Figure 5C). Interestingly, and contrary to the CDR1,2/MHC interaction, the energy profiles resulting from the center of the binding site (CDR3/peptide) efficiently discriminated the primary minimum from the secondary minimum, defining clearly which energy minimum leads to the native orientation.

Importantly, the typical shape of TCR rotation profiles carries information about the location of the native and the opposite minimum, as well as the location of orthogonal forbidden binding orientations. This seems to be exclusive to TCR rotation profile, as confirmed by CD8 and JAK2 rotation profiles that were calculated as negative controls (Figure 8). By rotating the CD8 protein in front of the pMHC, we observe a symmetrical signal, which can be explained by the homodimeric nature of the co-receptor. The JAK2 profile confirmed that a randomly selected monomeric structure does not reproduce similar energetic properties upon rotation.

### Outliers and Limitations

Clearly, the simplified rigid approach was not suitable for a number of crystal structures, such as MHC bearing long peptides in bulged conformations. Indeed, without a relatively flat binding surface, residues that are deeply buried upon binding might still be in contact with TCR after a 8Å unbinding translation. Therefore we restricted our test set to structures with a peptide not longer than 11 residues. The outlier structures 3mv8 and 2ol3 were treated separately, and the energy decomposition allowed the identification of a few outlier residues. Rotation profiles were then re-calculated (similar to Figure S3), and the identification of primary minima was made possible. In the case of MHC class II molecules, the length of the peptides was not an issue since longer peptides do not adopt a bulged conformation as it is observed in MHC class I.

In general, at distances smaller than 8Å from the pMHC, a large amount of steric clashes occurs during the TCR revolution. Furthermore, the orientation and final binding mode of the TCR is then governed by the short range atomic details, the non-polar interactions and desolvation, and the induced fit of the binding sites. The computation of the effective energy variations at such small distances is out of the scope of this study.

### Outlook

Crystal structures represent a considerable interest for the field of molecular modeling, as illustrated by the numerous studies of the TCRpMHC binding [36]. Molecular modeling studies on these structures are performed notably to understand the binding process of the TCR [37], the effect of mutations [38] and to perform *in silico* protein engineering [39]. Since 1996 and the first determination of the structure of the TCRpMHC complex [2], subsequent releases have revealed the variety of TCR binding orientations depending altogether on the antigen epitope, the MHC and the α/β pairing of the TCR [5].

Despite the increased number of available TCRpMHC crystal structures, modeling has quickly become an important complementary approach as experimental techniques allow very quick sequencing of entire TCR repertoires. Molecular modeling methods tried to address *in silico* the question of TCR binding mode prediction in various ways, including manual orientation based on experimental data [40], homology modeling [41] [7], or protein-protein docking (private communication). Recently, the study from Roomp *et al.* [4] presented an algorithm dedicated to TCR/pMHC interaction that quite reliably predicted the contacts between the pMHC and the CDR loops, using a training set of existing crystal structures, paving the road for *in silico* TCR binding mode prediction. Promising approaches are alternatively based on recent progress in identifying experimentally the buried surface footprint upon protein-protein complexation. The approach by Varani *et al.* [9] successfully identified the TCR footprint on pMHC by NMR chemical shift mapping.

The results of the present work showed a surprisingly good agreement between the primary minimum of rotation profiles at 8Å and the native orientation of the TCR bound to pMHC. We demonstrated a good robustness of the results upon TCR structural variations seen in Molecular Dynamics simulation. Furthermore, we also observed that rotation profiles of long-range interactions do show a relevant signal when unbound TCR crystal structures are put in front of the target pMHC, and that perfect shape complementarity is not required. As most important CDR loops shifts between the unbound and the bound structures were recorded on CDR3 [27], we found the result consistent with our energy decomposition showing that the long range signal is mostly carried by the outside of the binding site. These results investigated the possibility to predict the TCRpMHC binding mode orientation in a pure *in silico* approach, through computation of long-distance interactions.

To our knowledge, in a TCRpMHC modeling process [7], the precision that is required on TCR orientation over pMHC for fine interface and contact refinements is ±10° (data not shown), close to the 11° average distance between the native and predicted orientations using the present approach. Although the precision that was provided by the rigid body simplification may not be satisfying for the prediction of exact binding modes, the quick execution of the protocol makes it well suited for a preliminary search of TCR orientation, which is indeed already defined prior to the binding process.

We demonstrated the potential of this approach as a potential component in a TCRpMHC structural modeling pipeline, by searching the binding mode of the A6 TCR as an average of the rotation profile minima obtained after modeling TCR by homology. As mentioned in the results section, we obtained an average deviation of 12.2° (SD = 16.0) from the native orientation. Typical approaches for TCRpMHC structure predictions make use of homology modeling of the complex, complemented by *ab initio* refinement of CDR loops at the interface with pMHC [7]. It has been stated that the prediction of the TCR binding orientation was indeed an issue in the process, preventing the correct contact predictions in case of false binding [7]. The present study provides data on how rotation profiles could be used to guide this critical step in homology modeling. The reliability of such approaches will be assessed elsewhere. The approach could also easily be extended to the generalized approach of protein-protein docking after the areas of binding sites have been correctly identified.

## Supporting Information

Figure S1 **TCR rotation profiles of the MHC class II test set.** The polar contribution to the effective energy of the TCRpMHC complex is plotted against TCR rotation angle around the x axis, after an 8Å translation away from the pMHC. Positions that make steric clashes are ignored.
(EPS)

Figure S2 **GB-MV2 electrostatic solvation energy variation of a single TCR, upon rigid rotation in Cartesian space, calculated with CHARMM.** pMHC is not present. The amplitude of the unphysical energy variation, which comes from the mathematical grid-based description of the system, is larger than 15 kcal/mol and makes the approach unsuited for the computation of TCR rotation profiles.
(EPS)

Figure S3 **Contribution of the MHC-helices and CDR1,2 to the TCR rotation profiles of the test set.** The polar effective energy of the sub-system is plotted against TCR rotation angle around the x axis, after an 8Å translation away from the pMHC.
(EPS)
